# Unilateral endogenous fungal endophthalmitis after esophageal cancer surgery: a case report

**DOI:** 10.1186/s12879-018-3607-6

**Published:** 2018-12-18

**Authors:** Quan-Yong Yi, Wen-die Li, Li-shuang Chen, Zhi-Sha Bai

**Affiliations:** Department of Ophthalmology, Ningbo Eye Hospital, 855 Minan Road, Ningbo, 315040 China

**Keywords:** Endogenous, Fungal, Endophthalmitis, Postcancer surgery, Case report

## Abstract

**Background:**

This study reports a case of Unilateral Endogenous Fungal Endophthalmitis After Esophageal Cancer Surgery.

**Case presentation:**

One patient presented with a month-long loss of vision in his left eye, he had surgery for esophageal cancer 2 months earlier. The patient underwent cataract surgery (by phacoemulsification) in the left eye combined with 25-gauge vitrectomy and silicone oil tamponade. The microbiological culture pointed to infection with *Candida albicans*. At 3-month follow-up, the unaided visual acuity of left eye was 0.02 and corrected visual acuity was 0.2. In addition, there was no recurrence of the endophthalmitis within 1 year of the surgery.

**Conclusion:**

The early diagnosis of endogenous fungal endophthalmitis is difficult, and the disease is very likely to be misdiagnosed as uveitis. It is therefore critical to improve awareness of this condition and to reduce the incidence of its misdiagnosis.

## Background

Endogenous endophthalmitis is an infection due to fungi or bacteria that enter the eye through the circulating blood, causing inflammation of intraocular tissues such as the vitreous body and retina. Endogenous endophthalmitis accounts for 2 to 15% of all cases of endophthalmitis [1, 2]. Herein we report one cases of Unilateral Endogenous Fungal Endophthalmitis After Esophageal Cancer Surgery.

## Case presentation

**Fig 1:**
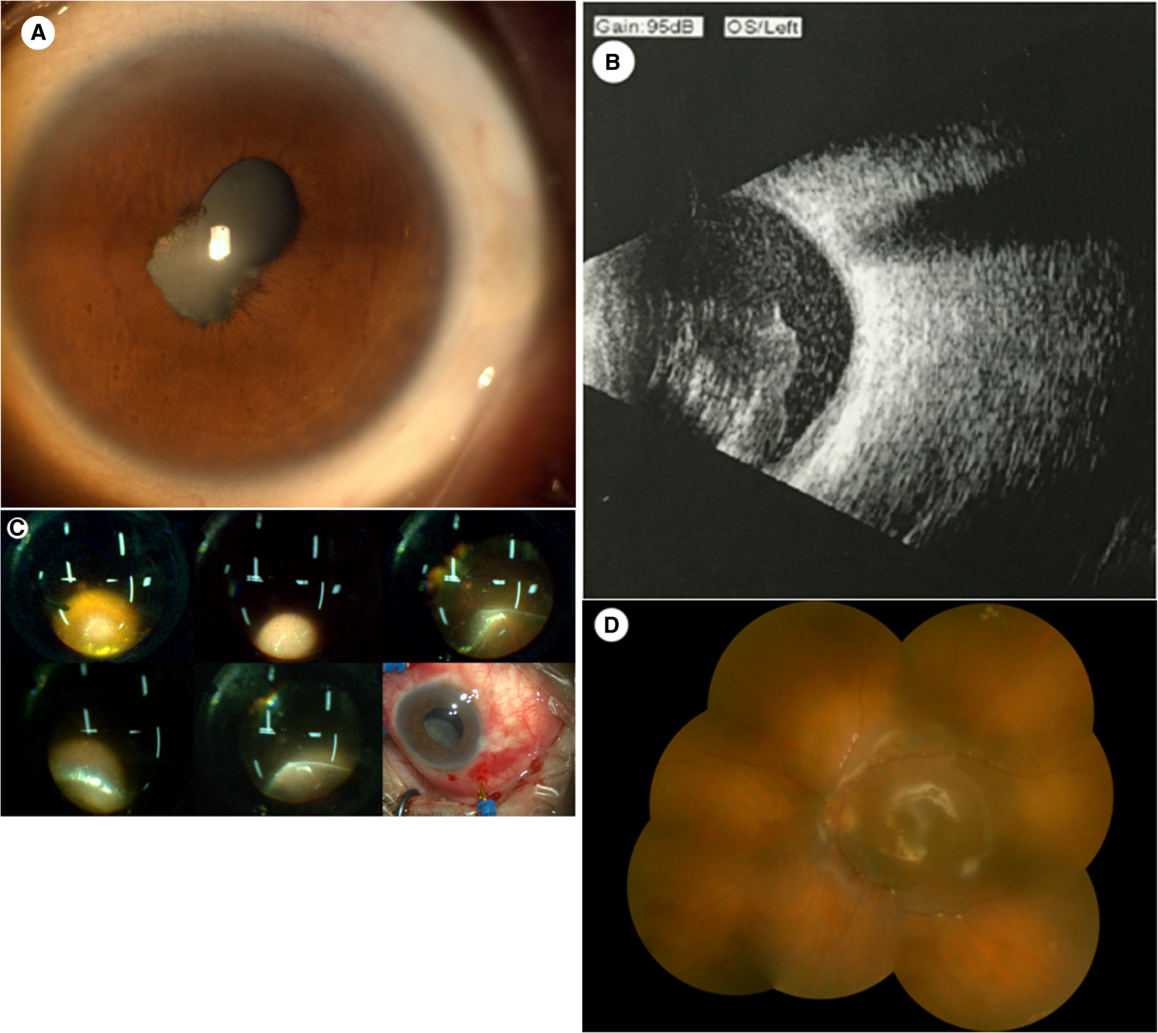
**a** Mixed conjunctival hyperemia, mild corneal edema, aqueous flare, posterior synechia, non-dilating pupil, and pigment deposits on the anterior lens capsule were observed in the left eye. **b** Pronounced vitreous opacity and macular retinal thickening was observed in the left eye. **c** A large amount of beaded or cheesy white exudates in the posterior pole and the peripheral retina was observed during the surgery. The lower right figure showed that the ciliary body was coated with a white film-like substance. **d** At the 1-month follow-up, the color fundus photograph showed that the vitreous cavity was filled with silicone oil, the retina was flat, and no white exudates was observed in retina

A 63-year-old man visited our hospital’s outpatient clinic on August 1, 2016, reporting a month-long loss of vision in his left eye; he was admitted with the tentative diagnosis of uveitis. He had been treated at another hospital a month earlier with no improvement, and his vision continued to deteriorate. As for the patient’s medical history, he had surgery for esophageal cancer 2 months earlier. The results of the eye examination were as follows: his visual acuity was only light perception in the left eye, respectively. In the left eye, we observed a transparent cornea, mild aqueous flare, partial posterior synechia, nondilating pupil, and pigment deposits on the anterior lens capsule (Fig. 1a). In addition, the part of lens behind the pupil was highly turbid, and the fundus could not be seen. B-scan ultrasonography showed pronounced vitreous opacities and macular retinal thickening in the left eye (Fig. 1b). On August 3, 2016, under local anesthesia, the patient underwent cataract surgery (by phacoemulsification) in the left eye combined with 25-gauge vitrectomy and silicone oil tamponade. During the surgery, we noticed vitreous opacities that looked like floccose white balls as well as flocculent vitreous opacities; part of the vitreous was therefore aspirated for bacterial and fungal culture. After the turbid vitreous body was removed, we observed a flat retina and a many beaded or yeast-like white plaques and spots in retina. We also found that the ciliary body was coated with a white film-like substance (Fig. 1c). So, a pus sample was collected from his vitreous body for routine microbial cultivation. After incubation for 24 h, cream white colonies without hemolytic reaction were detected. Gram staining showed the presence of Gram-positive spore morphology clusters. The strain was identified with YST card by Vitek 2 Compact system (bioMérieux, USA) as *Candida albicans*. In addition, antibiotic susceptibility tests were determined using the ATB™ fungns 3 kit (bioMérieux, USA), and the strain was found to be susceptible to fluconazole, itraconazole, amphotericin B, voriconazole, and 5-fluorocytosine. At the meantime, we had collected a sample for blood culture; after two days, the patient refused to collect another blood samples for culture again. So it’s very regret that we couldn’t obtained a positive result for blood culture. Subsequently the patient received antifungal treatment via an intravenous infusion comprising 0.1 g of fluconazole once daily for 3 days followed by the oral administration of 100 mg of itraconazole once daily for 1 month. At 3-month follow-up, the unaided visual acuity of left eye was 0.02 and corrected visual acuity was 0.2. The color fundus photograph showed that the vitreous cavity of the left eye was filled with silicone oil, the retina was flat, and there were no white exudates in retina (Fig. 1d). These results indicated a good recovery. In addition, there was no recurrence of the endophthalmitis within 1 year of the surgery.

## Discussion and conclusions

Endogenous fungal endophthalmitis is characterized by its low incidence, insidious onset, and high degree of destructiveness, which can lead to atrophy of the eyeball and permanent visual impairment. The risk factors for endogenous fungal endophthalmitis include systemic diseases (e.g., diabetes, liver cirrhosis, malignant tumors, and acquired immunodeficiency syndrome) and/or the presence of an infectious lesion (e.g., liver abscess, meningitis, and lung abscess) [2–4]. Among all cases of endogenous fungal endophthalmitis, endogenous *C. albicans* endophthalmitis is the most common type, representing 75 to 80% of the cases; *Aspergillus* is the second most common causative organism [5–7]. The effective treatment of endogenous fungal endophthalmitis rests on the combination of timely vitrectomy and systemic antifungal therapy. In addition, silicone oil has antimicrobial activity against *Staphylococcus aureus, Pseudomonas aeruginosa*, and *C. albicans* [8, 9]. The patient reported here, who suffered from endogenous *C. albicans* endophthalmitis, had the risk factor of a malignant tumor. Before we operated on his eye, the endophthalmitis had progressed to stage III (vitreous opacity), and the eye could only perceive light. Following timely surgery and antifungal treatment, we were able to restore partial visual function to the affected eye. In general, the early diagnosis of endogenous fungal endophthalmitis is difficult, and the disease is very likely to be misdiagnosed as uveitis. Moreover, with the improper use of steroids or immunosuppressive agents, endogenous fungal endophthalmitis can rapidly progress. In recent years, in the context of the increased use of intravenous drugs and the application of immunosuppressive agents, the incidence of endogenous fungal endophthalmitis has tended to increase. It is therefore critical to improve awareness of this condition and to reduce the incidence of its misdiagnosis.
